# Do COVID-19 Infections Result in a Different Form of Secondary Hemophagocytic Lymphohistiocytosis

**DOI:** 10.3390/ijms22062967

**Published:** 2021-03-15

**Authors:** Raymond Chu, Charmaine van Eeden, Sneha Suresh, Wendy I. Sligl, Mohammed Osman, Jan Willem Cohen Tervaert

**Affiliations:** 1 Division of Rheumatology, Department of Medicine, The Ottawa Hospital, University of Ottawa, Ottawa, ON K1H 7W9, Canada; rchu@toh.ca; 2 Division of Rheumatology, Department of Medicine, University of Alberta Hospital, University of Alberta, Edmonton, AB T6G 2R3, Canada; vaneeden@ualberta.ca (C.v.E.); mosman@ualberta.ca (M.O.); 3 Division of IHOPE, Department of Pediatrics, Stollery Children’s Hospital, University of Alberta, Edmonton, AB T6G 1C9, Canada; ssuresh@ualberta.ca; 4 Department of Critical Care Medicine and Division of Infectious Diseases, Department of Medicine, University of Alberta Hospital, University of Alberta, Edmonton, AB T6G 2B7, Canada; wsligl@ualberta.ca

**Keywords:** COVID-19, SARS-CoV-2, natural killer cells, cytokine storm, hemophagocytic lymphohistiocytosis, macrophage activation syndrome

## Abstract

The coronavirus disease 2019 (COVID-19) pandemic, caused by severe acute respiratory syndrome coronavirus 2 (SARS-CoV-2), has resulted in significant morbidity and mortality across the world, with no current effective treatments available. Recent studies suggest the possibility of a cytokine storm associated with severe COVID-19, similar to the biochemical profile seen in hemophagocytic lymphohistiocytosis (HLH), raising the question of possible benefits that could be derived from targeted immunosuppression in severe COVID-19 patients. We reviewed the literature regarding the diagnosis and features of HLH, particularly secondary HLH, and aimed to identify gaps in the literature to truly clarify the existence of a COVID-19 associated HLH. Diagnostic criteria such as HScore or HLH-2004 may have suboptimal performance in identifying COVID-19 HLH-like presentations, and criteria such as soluble CD163, NK cell activity, or other novel biomarkers may be more useful in identifying this entity.

## 1. Hemophagocytic Lymphohistiocytosis

Hemophagocytic lymphohistiocytosis (HLH) is a potentially life-threatening disorder characterized by uncontrolled activation of cytotoxic T cells, natural killer (NK) cells and macrophages which results in the hypersecretion of cytokines and immune-mediated organ damage [1,2]. Clinical presentation includes; fever, splenomegaly, coagulopathy, liver dysfunction, cytopenias, hypertriglyceridemia, hyperferritemia, hemophagocytosis, neurologic dysfunction and diminished NK cell activity [1,2,3]. It is classically subdivided between primary (familial or lymphoproliferative disorder) and secondary (acquired) HLH, affecting children and adults, respectively.

Familial HLH is divided into four subtypes based on the gene affected by mutation [4]. Mutations of *PRF1*, *UNC13D*, *STX11* and *STXBP2* result in impaired granule mediated cytotoxicity by natural killer (NK) or cytotoxic T cells [4]. Effective cytotoxicity is essential for the control of infection, as well as regulation and termination of the immune response [5,6]. The PRF1 gene was the first to be associated with familial HLH, and accounts to up to 58% of familial HLH [7,8]. *PRF1* encodes perforin, lower levels of which result in decreased natural killer cell cytotoxicity and reduced effector cell lysis due to impaired pore formation [7,8]. The genes *UNC13D*, *STX11* and *STXBP2* encode vesicle priming and fusion proteins, the loss of which results in impaired cytotoxic granule exocytosis [9,10,11,12]. Lymphoproliferative disorders caused by mutations in BIRC4 or SHD21A are associated with an increased sensitivity to Epstein–Barr virus (EBV) infection, often leading to the development of HLH [13].

Secondary HLH is associated with hematological malignancies, viral infection, and rheumatic disease, with the latter being termed as macrophage activation syndrome (MAS)) [1,14,15,16,17]. Ramos-Casals et al. detailed 2197 adult HLH cases between 1974 and 2011 and identified HLH associated with an infectious trigger in 50.4%, malignant trigger in 47.7%, autoimmune trigger in 12.6% and an unknown trigger in 3.7%. Nearly one third of cases had more than one underlying cause [18]. Hematologic malignancies such as lymphomas, T/NK-cell disorders, acute leukemias, lymphoproliferative diseases, and myelodysplastic syndrome, are the most common triggers of malignancy derived HLH [1,14,19]. Interestingly, EBV is a frequent co-trigger affecting 24% of patients and 88% of patients during chemotherapy [19,20]. Chemotherapeutic triggers cell lysis and necrosis, resulting in a release of IL-5, IL-13, IL-10, IL-6, TNF-α, IFN-γ and IL-1β, further potentiated by tumor infiltrating lymphocytes [21,22]. Chemotherapy can also increase underlying single nucleotide polymorphism or subclinical mutation leading to development of HLH [21,23,24].

Macrophage activation syndrome is a subset of secondary HLH in the context of autoimmune disease, with systemic lupus erythematosus most frequently reported, followed by adult-onset Still’s, rheumatoid arthritis and vasculitis [18]. Pathogenesis of MAS is attributed to a patient’s underlying immune activity with studies showing elevated pro-inflammatory cytokines, most notably IL-6, IL-1β and IL-18 [25,26,27]. Genetic predisposition and a potential infectious triggers can initiate the development of macrophage activation, resulting in the expansion and activation of T cells, particularly CD8 cytotoxic T-cells [1]. Activated T-cells release IFN- γ, that in turn further stimulates activation of macrophages [1]. Natural killer cell dysfunction also contributes to this process in that deceased function has both an effect of reduced ability to remove viral triggers and reduced immunomodulatory effect on CD8 T-cell IFN-γ expression [15,28,29]. NK cells are divided into CD56^BRIGHT^ and CD56^DIM^ subsets, with CD56^DIM^ demonstrating higher cytotoxic activity and perforin expression. On the other hand, CD56^BRIGHT^ cells demonstrate higher cytokine production (notably IFN-γ, TNF-β, granulocyte macrophage-colony-stimulating factor, IL-10 and IL-13), playing a more immunoregulatory role and only becoming cytotoxic when activated [30,31]. While the relationship between CD56^BRIGHT^ and CD56^DIM^ is controversial, the CD56^BRIGHT^ NK cell subset is diminished in both MAS and HLH, as is cytotoxicity [32,33].

Epstein–Barr virus (EBV) is a ubiquitous virus that infects nearly all people worldwide without serious sequelae. It is however recognized as the leading cause of infection-associated HLH, with other herpes viruses also playing a prominent role. Without early and effective therapy, EBV-HLH has a high mortality rate, frequently due to multiorgan failure. EBV-HLH can result from (1) HLH development secondary to EBV infection, in apparently EBV-immune individuals, (2) EBV infection where there is a genetic predisposition for lymphoproliferative disease (BIRC4 or SHD21A), (3) chronic active EBV infection, and (4) aggressive NK cell leukemia, and T/NK cell lymphoma [34,35,36].

Influenza viruses have also been associated with the development of HLH; influenza B, H1N1, H5N1, H3N2 have all seen cases of HLH, though the presentation is often varied with ineffective cytotoxicity, elevated ferritin levels, thrombocytopenia and in rare instances the development of lethal multi-organ failure syndrome being observed [37,38,39]. Ebola virus infection has been shown to bare striking resemblance to HLH and MAS, with the presence of fever, cytopenia, hypofibrinogenemia, low NK cells levels and in particular hyperferritinemia which was shown to be associated with hemorrhage and death [40,41]. Although rare in occurrence, human immunodeficiency virus (HIV) should also be considered when the cause of HLH is not apparent [42]. While less frequent, bacterial, parasitic and fungal infections have also been reported to trigger infection-associated HLH [18]. Infection-associated HLH have been demonstrated to have elevated IFN-γ, s-IL-2r, IL-6, IL-10 and IL-18, as well as higher frequencies of CD8 T cells suggesting similar profiles to MAS [43,44]. Studies comparing non-infectious versus infectious causes of HLH have demonstrated significantly lower mortality rates in the infectious cohorts [45,46].

## 2. Clinical Criteria for HLH

As the clinical features of HLH frequently mimic septic shock syndrome, the diagnosis of HLH, particularly in adults, is challenging and prompt verification is pivotal to ensure early and rapid treatment in this rapidly progressing disorder [47,48,49]. Though the first reported case of HLH was described in 1952, the diagnostic guidelines for HLH were first put forth 39 years later by the familial hemophagocytic lymphohistiocytosis (FHL) study group of the Histiocyte Society, which comprised of five criteria including fever, splenomegaly, bicytopenia, hypertriglyceridemia (≥3.0 mmol/L) and/or hypofibrinogenemia (≤1.5g/L), and hemophagocytosis [50,51]. In 1994 the first prospective international treatment protocol (HLH-94) was introduced [50]. These guidelines were updated in 2004, which expanded on the existing five criteria, adding low or absent NK cell activity, hyperferritinemia (≥500 µg/L) and elevated soluble CD25 (soluble IL-2 receptor [sIL-2r]) (≥2400 U/mL). Five of the eight criteria are required to make the diagnosis of HLH, unless patients have a molecular diagnosis of an underlying genomic defect known to cause FHL [8,52].

While the HLH-2004 criteria were widely used, limitations existed in that they were proposed in the context of pediatric populations and did not differentiate primary HLH from secondary disease. As such, the HScore was developed and validated in the diagnosis of reactive HLH in 2014. The HScore incorporated nine variables, including features of immunosuppression, fever, organomegaly, hypertriglyceridemia, hyperferritinemia, aspartate transaminase (AST), hypofibrinogenemia, cytopenias and presence of hemophagocytosis on bone marrow aspirate. Scores range between zero and 337, with the probability of HLH being <1% with an HScore of <90 points and >99% with an HScore of ≥241 points. An HScore of 169 has a reported sensitivity of 93%, a specificity of 86%, and accurate classification of 90% of patients [53].

The clinical and laboratory findings utilized by the HLH-2004 and HScore are reflective of the proinflammatory state that is the hallmark of HLH. Fevers are a result of high concentrations of IL-1 and IL-2, with macrophage and T-cell proliferation resulting in organomegaly [1,54]. Cytopenias are caused by cytokine-mediated inflammation, particularly involving high levels of TNF-α and IFN-γ [55]. Hypertriglyceridemia is a consequence of TNF-α secretion and its inhibitory effects on lipoprotein lipase, which hydrolyzes triglycerides for use by muscle and adipose tissues [56,57]. Higher triglyceridemia is noted in HLH patients compared to patients with sepsis [58]. Ferritin is an iron storage protein and an acute phase protein triggered by the release of IL-1 [59]. Increasing autophagy associated with HLH degrades ferritin and increases cellular iron, accumulating reactive oxygen species leading to cell death, also known as ferroptosis [60]. While HLH-2004 criteria established a ferritin cut off of ≥500 mg/L, the HScore criteria utilizes a higher lower limit cut off of <2000 mg/L, with additional points for ranges ≥2000 and ≤6000 mg/L, as well as >6000 mg/L [52,53]. While ferritin is an acute phase reactant, its concentration in sepsis is notably lower compared to those in secondary HLH, vasculitis and other rheumatic diseases [58]. Fibrinogen is also an acute phase reactant often elevated in sepsis, but is low in HLH. Hypofibrinogenemia in HLH is caused by plasminogen activators released by activated macrophages, increasing plasmin concentration, leading to increasing cleavage of fibrinogen [61]. Hypofibrinogenemia must be interpreted in the context of platelet count and coagulation profiles, which may suggest a component of disseminated intravascular coagulation, a known complication of HLH that is associated with increased mortality [45,46,62].

Due to the overlap in clinical features between sepsis and HLH, HLH is often undiagnosed in patients who are affected by both conditions, an occurrence that is not unusual in viral infection [47,49,63]. The treatment strategies for these conditions differ, and an accurate diagnosis is essential in critically ill patients. Multiple studies have suggested that reducing the number of diagnostic criteria for HLH may lead to earlier diagnosis and improved prognosis [63,64,65].

## 3. NK Cell Activity, Soluble IL-2 Receptor and Soluble CD163

While the diagnosis of HLH (through the HScore or the HLH-2004 criteria) can be made without biomarkers such as NK cell activity, s-IL-2r and soluble CD163, various studies (Table 1) have demonstrated the utility of decreased NK cell activity, increased s-IL-2r levels and increased soluble CD163 levels in primary and secondary HLH. As a result of these studies, s-IL-2r and decreased NK cell activity were added to the HLH-2004 criteria.

**Table 1:** Summary of published findings for NK activity, soluble IL-2 receptor and CD163 in primary and secondary causes of HLH.

	Decreased NK Cell Activity	Soluble IL-2 Receptor (Soluble CD25)	CD163
Primary	Perez et al. 1984 [66] Sullivan et al. 1998 [67] Kogawa et al. 2002 [68] Schneider et al. 2002 [69]	Komp et al. 1989 [70] Imashuku et al. 1995 [71]	Gao et al. 2019 [72]
Malignant	Konjević et al. 1999 [73]	Bien and Balcerska 2008 [74] Zhang et al. 2011 [75] Tsuji et al. 2014 [76] Hayden et al. 2017 [77]	Sadaat and Jang 2018 [78]
Infectious	National Research Project for SARS, BG. 2004 [79] McElroy et al. 2019 [80] Cimini et al. 2017 [81] Guo et al. 2011 [82] Schulert et al. 2016 [83]	Chellapandian et al. 2013 [84] McElroy et al. 2019 [80] Hofmann et al. 1991 [85]	McElroy et al. 2019 [80] Beltran et al. 2014 [86] Burdo et al. 2011 [87] Wiedemann et al. 2020 [88] Hasegawa et al. 2013 [89]
Autoimmune	Grom 2004 [15] Vastert et al. 2010 [90]	Bleesing et al. 2007 [91] Hayden et al. 2017 [77] Witkowska et al. [92]	Bleesing et al. 2007 [91] Sakumura et al. 2018 [93] Nishino et al. 2019 [94]

The primary functions of NK cells include their ability to eliminate virally infected cells, as well as their role in downregulating the systemic inflammatory response by killing activated inflammatory dendritic cells, monocytes, and T cells [95,96]. Decreased NK cell numbers and effector functions are associated with a multitude of conditions, including ageing, atherosclerosis, autoimmunity, hematologic malignancies and viral infection [81,82,97,98,99,100,101,102]. Decreased NK cell activity has been established in both primary and secondary forms of HLH [15,29,66,67,68,69,73,79,80]. Zhang et al. developed a flow cytometry-based assay for the detection of NK cell activity and evaluated 113 HLH patient and 64 healthy volunteer samples. They found that the mean NK cell activity in HLH was significantly lower than in healthy controls (20.23 ± 4.12%), and that primary HLH (10.76 ± 2.54%) could also be distinguished from secondary HLH (15.01 ± 3.62%) [103].

In the context of infection-associated HLH, decreased NK cell activity has been demonstrated in infections including, but not limited to, EBV, CMV, Ebola virus and in severe acute respiratory syndrome-related coronavirus (SARS-CoV-1), the causative pathogen in the 2002–2004 SARS outbreak [29,43,79,80]. NK cell activity is decreased in HLH, particularly with regard to loss of both cytolytic activity as measured by degranulation, and by IFN-γ production [15,69], the importance of which is highlighted by the fact that patients with influenza and systemic juvenile idiopathic arthritis that developed severe HLH more frequently carry mutations in genes implicated in NK cell cytotoxicity [83,90]. The interlekin-2 receptor (IL-2R, CD25) is primarily secreted by activated T-helper lymphocytes, but is also expressed by B cells, granulocytes and NK cells [104,105,106], the release of soluble IL-2r (sIL-2r) reflecting the activation of the immune system [70,71,74,75,76,77,91]. An interesting feature of sIL-2R is its ability to bind IL-2 with an affinity similar to that of the form present on the cell surface, suggesting it may have an immunosuppressive function [92,107]. sIL-2r is considered a reliable biomarker for disease activity in inflammatory disorders, and is included in the HLH-2004 criteria. In the HLH-2004 criteria, a cut off of ≥2400 U/mL was created for the diagnosis of HLH. In children this cut-off is reported to be 93% sensitive for HLH and is more sensitive than elevated ferritin [52]. Unfortunately, elevated sIL-2r can also be seen in autoimmune diseases, malignancies, viral infections (such as HIV and Ebola), as well as cardiac damage [76,80,91,108,109], which means adults levels are likely to be higher. sIL-2r is elevated in primary HLH, MAS and septic shock. In HLH the medians have been reported to be consistently higher than the proposed cut off, with medians ranging between 2963 and 21,500 U/mL [58,84]. This is in contrast to sepsis in which medians range between 3000 and 4250 U/mL [84]. In adults there is some overlap in sIL-R2 levels between HLH and sepsis and in particular lymphoma when considering a cut off of ≥2400 U/mL. Hayden et al. noted that the cut-off best optimizing specificity and sensitivity in diagnosing HLH is 2515 U/mL (sensitivity, 100%; specificity, 72.5%) and a cut off of >10,000 improved specificity to 93% [77].

CD163 is a cortisol-regulated monocyte and macrophage specific surface glycoprotein, the extracellular portion of which circulates in blood as a soluble protein (sCD163) [110]. Inflammatory stimuli trigger sCD163 cleavage and subsequent systemic release from monocyte/macrophage cell membranes, signaling macrophage activation [94,110]. sCD163 has been linked to obesity, sepsis, insulin resistance and type 2 diabetes mellitus [111]. sCD163 has been investigated as a biomarker for infection-associated HLH and MAS [72,91,93,94,112], though it is not included in HLH-2004 criteria or HScore. Elevated sCD163 has been linked to disease severity and mortality in viral infections of Ebola, HIV and Influenza and intriguingly these viruses are also associated with the development of HLH [80,86,87,88,89]. Levels of sCD163 were measured in severe sepsis patients by Feng et al. which showed a peak median of 4190 ng/mL on day 5 of intensive care unit admission, Bleesing et al. showed sCD163 levels in patients diagnosed with autoimmune-associated MAS to be 23,000 ng/mL, and Sadaat and Jang, indicated a level of 6384 ng/mL in a malignancy-related MAS patient [78,91,113]. Several studies have also compared directly the sCD163 levels seen in septic patients, with or without HLH. These studies demonstrated that septic patients who meet HLH-2004 criteria, have higher sCD163 levels compared to septic patients not meeting HLH-2004 criteria [112,114]. Further Gao et al. provided evidence that sCD163 could be used to distinguish primary HLH from MAS [72]. The study by Cui et al., however, found that the area under the ROC curve (AUC) for ferritin combined with sCD163 was superior to the AUC for either ferritin or sCD163 for distinguishing sepsis-associated hemophagocytic lymphohistiocytosis from sepsis [114].

## 4. COVID-19: Secondary HLH, Sepsis or HLH-Sepsis Overlap?

Coronavirus disease 2019 (COVID-19) is a disease caused by the severe acute respiratory syndrome coronavirus-2 (SARS-CoV-2), of which to date there have been over 25 million infections and over 800,000 deaths [115]. Severe COVID-19 shares distinct clinical and laboratory features with the four entities termed “hyperferritinemic syndromes”, including macrophage activation syndrome (MAS), adult-onset Still’s disease (AOSD), catastrophic anti-phospholipid syndrome (CAPS) and septic shock [116,117,118]. One of the most prominent features of SARS-CoV-2 infection [119,120,121] is the presence of a severe inflammatory signal resulting in a cytokine release syndrome ranging from pyrexia to severe clinical manifestations such as multi-system organ failure, acute respiratory distress syndrome (ARDS), and death [117,122,123,124]. Excessive cytokine release has been described in up to 20% of COVID-19 cases, and is reminiscent of the ARDS and secondary HLH observed in patients with SARS-CoV-1 and MERS-CoV [123,125,126]. Older age, comorbid conditions, elevated body mass index, lymphopenia, and elevated levels of C reactive protein (CRP), lactate dehydrogenase (LDH), d-dimer, ferritin, and sIL-2r have been associated with intensive care unit (ICU) admission and death [121,124,125,127,128,129,130]. COVID-19 patients display high levels of both inflammatory cytokines and chemokines (IL-1a/β, IP-10, MCP-1), with severe cases showing elevation in TNFα, IL-1, IL-6, IL-18, IL-8, IL-10, MCP-1 and MIP-1A [119,124,127,131,132]. These findings highlight the possibility of an HLH-sepsis overlap triggered by COVID-19. Furthermore, a high incidence of thrombotic complications has been described in ICU patients [133,134,135], on average approximately 30% of these patients developed venous or arterial thromboembolic disease which can progress to potentially lethal disseminated intravascular coagulation (DIC). Coagulation is intricately linked with inflammation, in particular elevated levels of the pro-inflammatory cytokine, Interleukin 6 (IL-6) and C reactive protein (CRP) are known to downregulate natural anticoagulants, the resultant increase in coagulation in turn further increases inflammatory responses [136]. Intriguingly, it has been suggested that secondary CAPS may be involved in the development of thrombosis in COVID-19 patients [137], and indeed lupus anticoagulant (LA), antiphospholipid (auto) antibodies (aPL-Abs) and β2 glycoprotein 1 (β2-GP1) autoantibodies have been identified in COVID-19 patients [138,139,140,141]. David and Shoenfeld, also highlighted that the anosmia suffered by a high percentage of patients [142,143,144], could be related to autoimmunity [145].

Differentiating sepsis from an HLH-sepsis overlap syndrome is difficult in that features of HLH can often be found in patients with sepsis, and severe sepsis has been shown to be an early manifestation of HLH in multiple studies [63,64]. Due to the radically different treatment approaches, early diagnosis and prompt immunosuppressive treatment are vital for favorable patients’ outcomes. Currently, with no effective therapy for severe COVID-19, being able to identify a subset of patients with an HLH-sepsis overlap syndrome would support the need for further studies investigating the possible benefit of immunosuppressive therapies. The need for caution is, however, emphasized, in particular considering the experience gleaned from the 2002–2004 SARS outbreak. The SARS-CoV-1 virus was highly aggressive, with around 20–30% of patients requiring intensive care and an overall fatality of approximately 15% [146,147]. The SARS outbreak ultimately affected more than 8000 patients and caused 774 deaths in 26 countries, with its lethal nature prompting early attempts at treating severe disease with immunosuppressive agents such as antiviral agents including ribavirin, protease inhibitors, intravenous immunoglobulins (IVIg), interferon alpha and corticosteroids. While SARS was relatively short-lived, ultimately negating the need for a globally distributed vaccine, retrospective analyses of these treatment methods showed inconsistency in the treatments for SARS-CoV-1. Studies analyzing these treatments were later shown to be inconclusive and even had possibly harmful effects [148].

Mildly symptomatic SAR-CoV-2 infections generally cause a release of pro-inflammatory cytokines at lower levels than severe disease which is self-limited as a defense response is mounted against the virus. Use of dexamethasone, a generalized anti-inflammatory agent, remains the intervention with the strongest evidence of efficacy in such patients [149]. In patients with severe disease, loss of immune mediation and subsequent cytokine storm leads to initial lung epithelial damage, ARDS and other organ damage, with end stage disease increasingly being recognized as an endothelial injury with subsequent widespread vascular leakage and thrombosis; a process mediated by IL-1, IL-6 and TNF-α [150,151]. IL-1 induces leukocyte adhesion and reduces VE-cadherin production, impeding endothelial barrier function leading to vascular leakage [150,152]. IL-1 also promotes IL-6, which increases fibrinogen and decreases plasminogen activator inhibitor-1, promoting a prothrombotic, antifibrinolytic environment [151]. Increasing concentrations of TNF-α has been shown to induce depolymerization of F-actin, an important component of the cytoskeleton, to monomeric G-actin, compromising the endothelial barrier and stimulating intercellular gap formation [153]. Naturally, given this pathophysiology of progression to severe disease, targeted immunosuppression has become of great interest in COVID-19.

New studies continue to be published for targeted immunosuppression including IL-6 inhibition with tocilizumab and IL-1 inhibition with anakinra. As in the case of tocilizumab, we continue to see varying results, with an early retrospective cohort study suggesting possible reduction in risk of invasive mechanical ventilation or death in severe COVID-19 patients and a subsequent randomized, double-blind, placebo-controlled trial suggesting tocilizumab as not effective in preventing intubation or death in moderately ill COVID-19 patients, and being unable to rule out possible harmful effects [154,155]. Similarly, anakinra was demonstrated to be associated with clinical improvement in non-ICU, COVID-19 patients with ARDS in a retrospective cohort and demonstrated to reduce need for invasive mechanical ventilation in the ICU and mortality among patients with severe COVID-19 in a prospective cohort study [156,157]. A recent randomized controlled trial, however, has shown that anakinra did not improve patient outcomes in patients with mild to moderate COVID-19 pneumonia [158].

While tocilizumab and anakinra have shown varying results, with randomized trials not showing efficacy, these medications have been shown to be effective in the treatment of secondary HLH, with tocilizumab being effective in drug-induced HLH, while anakinra is the treatment of choice in macrophage activation syndrome [26,159]. Other treatments in secondary HLH include non-targeted immunosuppression with IVIg, as well as B-cell depletion with rituximab, particularly in EBV or other virus-related HLH [160]. IVIg is thought to have an immunomodulatory effect by inhibiting complement activation and neutralizing cytokines. While IVIg has not been robustly studied in COVID-19, a similar mechanism is proposed in the use of convalescent plasma acquired from individuals who had recovered from COVID-19 in the treatment of COVID-19 patients, with the thought that, in addition to the immunomodulatory effect of immunoglobulins, that existing antibodies would act to neutralize the virus. Unfortunately, convalescent plasma has been shown to be ineffective in multiple randomized controlled trials, and the presence of anti IFNα autoantibodies in survivors of severe COVID-19 may result in an unwanted decrease in host antiviral response [161,162,163]. Lastly, the use of rituximab in the treatment of EBV-related HLH has been established and is thought to be effective in that EBV resides in B-cells and subsequent B-cell depletion reduces EBV load and inflammatory response [84]. Perceived problems for rituximab in COVID-19 patients are twofold. Firstly, COVID-19 is not known to reside within B-cells. Secondly, B-cell depletion has the potential to impact antibody formation against SARS-COV-2, whether it be through natural acquisition or through vaccine administration [164].

Beyond targeted immunosuppression, drugs that target upstream mechanisms are of interest in COVID-19. Phosphodiesterase inhibition (PDEi) has been proposed as a potential treatment option for COVID-19 infection, as phosphodiesterase enzymes decrease cyclic guanosine monophosphate (cGMP) and cyclic adenosine monophosphate (cAMP) and promote cytokines such as TNF-α. Inhibition of PDE induces inhibition of proinflammatory mediators (PDE4/5), deceleration of lung fibrotic changes (PDE4), and inhibition of platelet aggregation (PDE3) [165]. To our knowledge, PDEi has not been evaluated in HLH and its role in secondary HLH is unknown, although mutations of the perforin gene involving cyclic nucleotide phosphodiesterase activities has been identified in familial HLH [166]. Another drug of interest in COVID-19 infection is fingolimod, a non-selective agonist of sphingosine 1-phosphate receptor, known to inhibit IL-1α, IL-1β, IL-6, IL-10, MCP-1, TNF-α and GM-CSF [167]. Similarly to PDEi, fingolimod has not been evaluated in HLH, and interestingly, has been reported to not preclude the development of HLH secondary to HSV-2 [168].

Several studies have been published to date that identify clinical features in severe COVID-19 patients such as elevated IL-2, IL-6, TNF-α and IFN-γ, that can resemble the cytokine profile seen in HLH [169,170,171]. On the other hand, NK cell activating cytokines (IL-12, IL-15, and IL-21), usually elevated in HLH, have been shown to be lower in severe COVID-19 patients [130]. However, to identify and differentiate a true HLH-sepsis overlap secondary to COVID-19 from COVID-19 triggered septic shock absent HLH, additional biomarkers would be required.

The diagnosis of HLH, whether utilizing the H-Score or HLH-2004 criteria, is made based on clinical and laboratory findings characterized by markedly elevated ferritin, splenomegaly, cytopenias, hypofibrinogenemia, and elevated triglycerides. In adults, these criteria are not always met, and a more tailored reduced criterion may be of benefit [63,64,65]. This is evident in COVID-19 patients, where although an excessive cytokine reaction is evident, many of them do not fulfill the HLH criteria. A prospective study by Lorenz et al. assessed HScores and a modified HLH-2004 criterion in 19 severe COVID-19 patients admitted to ICU, found a median HScore of 122, in the absence of confirmed presence of hemophagocytosis on bone marrow aspirate and a median HScore of 157, if assumed positive bone marrow aspirate criterion [172]. Notably, Prilutskiy et al. performed autopsies on four confirmed COVID-19 patients which showed evidence of hemophagocytosis within pulmonary hilar/mediastinal lymph nodes but none of these patients showed hemophagocytosis in the bone marrow [173]. Of the 19 patients studied by Lorenz et al., only one patient reached a threshold >169 in the absence of the bone marrow aspirate criterion and six patients reached a threshold >169 if the bone marrow aspirate criterion was assumed positive [172]. Clark et al. retrospectively reviewed 152 patients with polymerase chain reaction confirmed COVID-19 patients and demonstrated a median HScore of 52, with the majority of patients not reaching sufficient thresholds for an HLH diagnosis, although notably, many variables were missing including 46 patients who did not have ferritin levels and no patients having bone marrow biopsies collected [174]. These studies raise the question of the utility of the HScore in COVID-19 patients and highlight a need for more specific, accessible means of identifying HLH.

As a result, more useful biomarkers present in HLH such as low NK cell activity and markedly elevated sIL-2r may be useful in identifying these patients [175]. Indeed, Osman et al. showed that that NK cell numbers are decreased in severe COVID-19 patients, and that CD56^DIM^ cells are reduced and CD56^BRIGHT^ cells elevated. The reduced cytolytic function was further emphasized by lowered expression of CD107a^pos^ cells [130]. They also showed that the significantly elevated levels of sIL-2r in these patients was inversely correlated with NK cell number. In addition, continued monitoring of these parameters, particularly hyperferritinemia and sIL-2r may be useful in evaluating response to therapies such as immunomodulation, and/or anti-viral therapies, as has been demonstrated in malignancy-associated HLH [74]. While soluble CD163 is not a feature of either the HScore or HLH-2004 criteria, its value as a diagnostic and prognostic biomarker in sepsis-associated HLH has been demonstrated in a different patient population [114]. Future studies investigating these parameters in severe COVID-19 patients are required to clarify the existence of a COVID-19-associated HLH and their utility in COVID-19.

## 5. Conclusions

Coronavirus disease 2019 is a disease that can lead to potentially life-threatening multi-organ failure with recent studies suggesting a cytokine storm, similar to the biochemical profile seen in HLH. As the diagnosis of HLH often includes non-specific clinical and laboratory findings, future studies including more useful biomarkers such as low NK cell activity, markedly elevated s-IL-2r and markedly elevated soluble CD163 may help to identify the existence of a COVID-19-associated HLH and may guide clinical trials using immunosuppressive therapy in these patients.

## Data Availability

No new data were created or analyzed in this study. Data sharing is not applicable to this article.

## Abbreviations

HLH	Hemophagocytic lymphohistiocytosis
NK	Natural Killer
MAS	Macrophage Activation Syndrome
IFN	Interferon
TNF	Tumour Necrosis Factor
EBV	Epstein-Barr Virus
CMV	Cytomegalovirus
FHL	Familial Hemophagocytic Lymphohistiocytosis
s-IL-2r	Soluble IL-2 Receptor
AST	Aspartate Transaminase
sCD163	Soluble CD163
